# The duration of pubertal growth peak among three skeletal classes

**DOI:** 10.1590/2177-6709.21.5.067-074.oar

**Published:** 2016

**Authors:** Waqar Jeelani, Mubassar Fida, Attiya Shaikh

**Affiliations:** 1 Resident Orthodontist, The Aga Khan University Hospital, Section of Dentistry, Department of Surgery, Karachi, Pakistan.; 2 Consultant Orthodontist/Associate Professor, The Aga Khan University Hospital, Program Director, Orthodontics Residency Program Section of Dentistry, Department of Surgery, Karachi, Pakistan.; 3 Consultant Orthodontist/ Assistant Professor, The Aga Khan University Hospital, Program Coordinator, Orthodontics Residency Program Section of Dentistry, Department of Surgery, Karachi, Pakistan.

**Keywords:** Puberty, Age of onset, Cervical vertebrae, Cephalometry

## Abstract

**Introduction:**

Pubertal growth peak is closely associated with a rapid increase in mandibular length and offers a wide range of therapeutic modifiability.

**Objective:**

The aim of the present study was to determine and compare the mean ages of onset and duration of pubertal growth peak among three skeletal classes.

**Methods:**

A retrospective cross-sectional study was conducted using lateral cephalograms of 230 subjects with growth potential (110 males, 120 females). Subjects were categorized into three classes (Class I = 81, Class II = 82, Class III = 67), according to the sagittal relationship established between the maxilla and the mandible. The cervical vertebral maturation stage was recorded by means of Baccetti's method. The mean ages at CS3 and CS4 and the CS3-CS4 age interval were compared between boys and girls and among three skeletal classes.

**Results:**

Pubertal growth peak occurred on average four months earlier in girls than boys (*p* = 0.050). The average duration of pubertal growth peak was 11 months in Class I, seven months in Class II and 17 months in Class III subjects. Interclass differences were highly significant (Cohen's *d* > 0.08). However, no significant difference was found in the timing of pubertal growth peak onset among three skeletal classes (*p* = 0.126 in boys, *p* = 0.262 in girls).

**Conclusions:**

Girls enter pubertal growth peak on average four months earlier than boys. Moreover, the duration of pubertal growth peak is on average four months shorter in Class II and six months longer in Class III subjects as compared to Class I subjects.

## RESUMO

**Introdução:**

o pico de crescimento puberal está intimamente relacionado a um rápido aumento no comprimento da mandíbula e propicia uma larga gama de abordagens terapêuticas.

**Objetivos:**

o objetivo do presente estudo foi determinar e comparar as idades médias ao começo do pico de crescimento puberal, bem como sua duração, nas três diferentes classes esqueléticas.

**Métodos:**

esse estudo retrospectivo transversal foi conduzido usando radiografias celafométricas laterais de 230 indivíduos com potencial de crescimento (110 homens, 120 mulheres). Os indivíduos foram categorizados em 3 classes (Classe I = 81, Classe II = 82, Classe III = 67), conforme a relação sagital presente entre a maxila e a mandíbula. O estágio de maturação cervical foi registrado por meio do método de Baccetti. As idades médias em EMVC3 e EMVC4, bem como o intervalo de idade EMVC3-EMVC4 foram comparados entre meninos e meninas e entre as três classes esqueléticas.

**Resultados:**

o pico de crescimento puberal ocorreu, em média, quatro meses mais cedo nas meninas do que nos meninos (*p* = 0,050). A duração média do pico de crescimento puberal foi de 11 meses nos indivíduos Classe I, 7 meses nos indivíduos Classe II e de 17 meses nos indivíduos Classe III. As diferenças interclasses foram altamente significativas (*d* de Cohen > 0,08). Porém, não foram identificadas diferenças significativas quanto à época de início do pico de crescimento puberal entre as três classes esqueléticas (*p* = 0,126 nos garotos, *p* = 0,262 nas garotas).

**Conclusões:**

as meninas entram no pico de crescimento puberal, em média, quatro meses antes dos meninos. Além disso, a duração do pico de crescimento puberal é, em média, quatro meses menor em indivíduos Classe II e seis meses maior nos indivíduos Classe III, em comparação aos indivíduos Classe I.

## INTRODUCTION

Modification of children's facial growth to achieve a more harmonious relationship between different facial structures is often part of orthodontic treatment.^1^ ^,^ ^2^ ^,^ ^3^ Normal human development is constituted of certain periods of growth accelerations and decelerations.^4^ ^-^ ^7^ The periods of rapid growth are of particular interest to orthodontists, as growth modifications are best achieved during the adolescent growth spurt when different facial bones are growing at a favourable rate.^4^ ^,^ ^5^ By initiating treatment at patient's optimal skeletal maturational stage, a favorable outcome with minimum risk of unwanted effects can be expected.^4^

Longitudinal studies based on lateral cephalograms have identified wide individual variations in the time of pubertal growth spurt onset and duration.^8^ In this context, identification of patient's maturation stage becomes a critical component of orthodontic diagnosis, helping to identify children of the same chronological age, but with different degrees of skeletal maturation.

Individual patient's skeletal maturity can be assessed by means of different biological indicators, for example, increase in body weight and height,^9^ ^-^ ^12^ skeletal maturation of the hand and wrist,^6^ ^,^ ^13^ dental development,^14^ sexual changes,^15^ ^,^ ^16^ and cervical vertebral maturation.^17^ ^,^ ^18^ ^,^ ^19^ Franchi et al^20^ reported several advantages of using the cervical vertebral maturation (CVM) method in assessing the skeletal maturity of an individual. These advantages include: straightforward appraisal of cervical vertebrae shape; more than 98% interexaminer reliability; and no need for second radiation exposure to determine patient's skeletal age.^20^ ^,^ ^21^

Several studies^4^ ^,^ ^19^ ^-^ ^24^ and a systematic review^25^ have established the CVM method as a highly reliable approach of assessing different stages of adolescent growth spurt. Current studies^26^ ^,^ ^27^ continue to establish that the CVM method can be used as an alternative to the hand and wrist radiographs to assess skeletal maturity. Cervical stage 3 (CS3) and cervical stage 4 (CS4) of the CVM method correspond to the initial and final stages of the accelerative portion of the pubertal growth peak, respectively.^4^ ^,^ ^24^ Longitudinal studies by Gu and McNamara^28^ as well as Perinetti et al^29^ report that the maximum increment in mandibular growth occurs between CS3 and CS4. The age interval between these two stages is regarded as the duration of the pubertal growth peak.^28^ ^-^ ^32^

A rapid increase in mandibular length during pubertal growth peak highlights the potential impact of variations in the time of pubertal growth peak onset and duration on the final size of the mandible.^28^ ^-^ ^33^ Thus, evaluation of such aberrations at the time of pubertal growth peak onset and duration may provide a better understanding of the development of different skeletal malocclusions and subsequently facilitate treatment of skeletal problems during this period of rapid growth.

The timing of pubertal growth peak varies significantly between males and females; thus, a separate analysis for girls and boys is highly desirable. However, previous studies failed to provide a comprehensive analysis of pubertal growth peak duration among three skeletal classes and reported combined results for male and female samples.^30^ ^,^ ^31^ ^,^ ^32^

In this context, this study was designed to determine and compare the mean ages of pubertal growth peak onset and duration among children with different skeletal classes.

## MATERIAL AND METHODS

A cross-sectional study was conducted at The Aga Khan University Hospital, Karachi. Ethical approval was obtained from the institutional Ethics Committee prior to data collection (3503-Sur-ERC-15). Sample size for three skeletal classes was calculated by taking α = 0.05 and keeping a power of study of 80%. Findings by Kuc-Michalska and Baccetti^30^ were used for sample size calculation, showing that a sample size of 63 in each group was sufficient in order to detect a clinically significant difference of 0.50 + 1.00 year in the mean age at CS4 between Class I and Class III subjects. In order to increase the power of study, the maximum number of available subjects was included in the study, which resulted in a total sample of 230 subjects.

This study was conducted on subjects of Pakistani origin and with growth potential (aged 9-17 years old). The following inclusion criteria were implemented: subjects with skeletal Class I, II or III relationships, normal vertical facial pattern (anterior cranial base to the mandibular plane angle = 32 + 5°, and lower anterior facial height to total anterior facial height 56 + 3%), and subjects in cervical stages CS3 or CS4 based on the CVM method.^4^ Subjects with history of orthodontic treatment, trauma or surgery to facial structures, any syndrome or developmental anomaly of facial structures, or any systemic disorder affecting growth were excluded.

**Figure 1:**
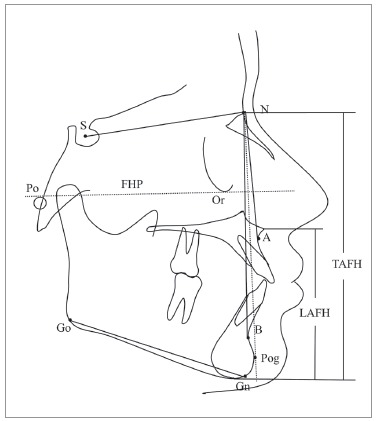
ANB angle and Downs facial angle (angle formed between FHP and NPog) were used to classify subjects into three skeletal classes. The SNMP angle (angle formed between SN plane and GoGn plane) and LAFH/TAFH ratio were used to determine the vertical growth pattern of a subject.

Patients' age was recorded to the nearest month and converted into decimal expression for further use in statistical analyses. Lateral cephalograms of all patients were traced manually on acetate paper by the main investigator, and the skeletal class of each subject was determined based on the ANB angle and Downs facial angle. The vertical facial pattern was assessed from the anterior cranial base to the mandibular plane angle (SNMP angle), and lower anterior facial height to total anterior facial height ratio (LAFH/TAFH) (Fig 1).^34^ ^,^ ^35^ Dental malocclusion was assessed on pretreatment dental casts. Subjects were divided into three groups, according to the following criteria:

» Skeletal Class I: subjects with ANB angle > 0° and < 5°; Downs facial angle > 83° and < 91°; and Class I molar relationship (81 subjects).
» Skeletal Class II: subjects with ANB angle > 5°; Downs facial angle < 83°; and more than half unit Class II molar relationship (82 subjects).
» Skeletal Class III: subjects with ANB angle < 0°; Downs facial angle > 91°; and more than half unit Class III molar relationship (67 subjects).

**Figure 2:**
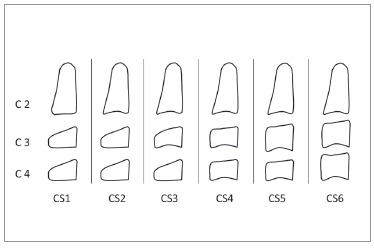
Cervical vertebral maturation stages according to Bacceti's method. CS3 is recognized by the appearance of a concavity in the lower margin of the body of C3 vertebra and either trapezoidal or rectangular horizontal shapes of C3 and C4 vertebral bodies. CS4 is identified by the appearance of a concavity on the lower margin of the 4^th^ cervical vertebra and rectangular horizontal shapes of C3 and C4 vertebral bodies.

Cervical vertebral maturation stages were assessed on the lateral cephalograms by means of Baccetti's method^4^ (Fig 2). The age interval between CS3 and CS4 stages was regarded as the duration of pubertal growth peak.^28^ ^-^ ^32^

Data were analyzed in SPSS for Windows (version 20.0, SPSS Inc. Chicago). The normality of variable age was assessed by means of Shapiro-Wilk test that showed normal distribution of data. The mean ages at CS3 and CS4 and the age interval between these two stages were compared between boys and girls by means of independent t-test. The mean ages at CS3 and CS4 and the age intervals between them were compared among three skeletal classes by one-way ANOVA and post-hoc Tukey tests. Effect sizes were calculated by means of Cohen's *d* and the recommended interpretations were used to describe the results.^36^ A *p* < 0.05 was taken as statistically significant, but this value was adjusted to the appropriate level when Bonferroni corrections were employed for multiple comparisons to minimize the chance of type I error. 

To test interexaminer reliability, 30 lateral cephalograms were randomly selected, and steps of tracing, landmark identification and measurement were repeated by the main investigator and a second observer. Kappa statistics was employed and showed that the values of coefficients of reliability were greater than 0.892 for the identification of skeletal class and the CVM stage.

## RESULTS

A total of 230 subjects (110 males and 120 females) were included in this study. The mean SNMP angle of the total sample was 31.28 ± 4.53°, and no significant difference (*p* = 0.065) was found among three classes. Similarly, the mean LAFH/TAFH of the sample was 55.37 ± 3.02%, and no significant difference was found among three skeletal classes (*p* = 0.125).

The mean ages at CS3 and CS4 were compared between the male and female samples by means of an independent sample t-test (Table 1). Significant sex-based differences in the mean ages at CS3 (*p* = 0.050) showed that the pubertal growth peak occurred around four months (0.33 years) earlier in girls, as compared to boys. The mean duration of pubertal growth peak was 11.7 months in girls and 13.3 months in boys. This sex-related difference in the duration of pubertal growth peak was found to be statistically significant, but had a small effect (Cohen's *d* = 0.13) (Table 2).

**Table 1:** A comparison of mean ages between boys and girls at CS3 and CS4.

Cervical stage	Boys (n = 110)	Girls (n = 120)	Mean difference	*p* value
	Mean ± SD (Years)	Mean ± SD (Years)	(Years)	
CS3	12.18 ± 0.81	11.85 ± 0.85	0.33 (3.9 months)	0.050*
CS4	13.30 ± 0.78	12.84 ± 0.92	0.46 (5.5 months)	0.004*

n = 230; SD: Standard Deviation; Independent sample t-test. *p < 0.05.

**Table 2:** Mean duration of pubertal growth peak in boys and girls.

	CS3	CS4	Duration of pubertal growth peak	CI inferior limit	CI Superior limit	Cohen's *d*	*p* value
	Mean ± SD (Years)	Mean ± SD (Years)	CS3 - CS4 (Years)	(Years)	(Years)		
Boys	12.18 + 0.81	13.30 + 0.78	1.11 ± 0.15 (13.3 months)	0.08	0.17	0.13	< 0.001
Girls	11.85 + 0.85	12.84 + 0.92	0.98 ± 0.17 (11.7 months)				

n = 230; SD: Standard Deviation; CI: Confidence Interval;

Cohen's *d* effect size: *low significance (0.2 - 0.5), **Moderate significance (0.5 - 0.8), ***High significance ( > 0.8).

Comparison of mean ages at CS3 and CS4 among three skeletal classes was performed by means of one-way ANOVA and post-hoc Tukey tests separately for the male and female samples (Table 3). No significant difference was found in the mean ages at CS3 in boys (*p* = 0.126) or girls (*p* = 0.262). However, highly significant differences (*p* = < 0.001) were present in the mean ages at CS4 among three skeletal classes for both males and females. 

**Table 3:** Comparison of mean ages at CS3 and CS4 among three skeletal classes.

	Cervical stage	Age (Years ± SD)			*p* value	Post-hoc Tukey HSD		
		Class I	Class II	Class III		I vs II	I vs III	II vs III
Girls	CS3	11.94 + 0.99	11.57 + 0.70	12.08 + 0.75	0.262	0.431	0.904	0.274
	CS4	12.95 + 1.04	12.26 + 0.56	13.62 + 0.43	<0.001*	0.003*	0.014*	<0.001*
Boys	CS3	12.22 + 0.83	11.93 + 0.89	12.47 + 0.61	0.126	0.492	0.613	0.103
	CS4	13.25 + 0.36	13.62 + 0.52	13.85 + 0.75	<0.001*	0.018*	0.015*	<0.001*

n = 230; SD: Standard Deviation; One-way ANOVA test. After applying Bonferroni correction for multiple testing. a *p*-value of <0.025 was taken as statistically significant. *p < 0.025.

The mean duration of pubertal growth peak was 0.95 ± 0.20 years (11.4 months) in Class I; 0.60 ± 0.15 years (7.2 months) in Class II; and 1.44 ± 0.16 years (17.3 months) in Class III children. The durations of pubertal growth peak were compared among various skeletal classes and showed that Class II subjects had on average 4.2 months shorter duration of pubertal growth peak, as compared to Class I subjects. On the other hand, the duration of pubertal growth peak was on average 5.9 months longer in Class III subjects, as compared to Class I counterparts. These interclass differences were characterized by highly significant effect (Cohen's *d* effect size > 0.08) (Table 4).

**Table 4:** Comparison of mean duration (in years) of pubertal growth peak among three skeletal classes.

	Duration of pubertal growth peak (CS3 - CS4 interval)	CI inferior limit	CI superior limit	Interclass difference	Cohen's *d*	*p* value
Class I (n = 81)	0.95 ± 0.20 (11.4 months)	-0.40	-0.29	-0.35 (4.2 months)	1.98***	< 0.001
Class II (n = 82)	0.60 ± 0.15 (7.2 months)					
Class I (n = 81)	0.95 ± 0.20 (11.4 months)	0.43	0.54	0.49 (5.9 months)	2.67***	< 0.001
Class III (n = 67)	1.44 ± 0.16 (17.3 months)					
Class II (n = 82)	0.60 ± 0.15 (7.2 months)	0.78	0.89	0.82 (9.8 months)	5.40***	< 0.001
Class III (n = 67)	1.44 ± 0.16 (17.3 months)					

Cohen's *d* effect size: *low significance (0.2 - 0.5), **Moderate significance (0.5 - 0.8), *** High significance (> 0.8).

## DISCUSSION

Variations in pubertal growth spurt onset and duration may affect the final size of different craniofacial structures.^10^ ^,^ ^37^ Longitudinal studies have shown that growth changes during adolescent growth spurt are more pronounced in the mandible, as compared to the maxilla.^10^ ^,^ ^38^ ^,^ ^39^ The current study reports that the onset of pubertal growth peak occurs around four months earlier in girls, as compared to boys. However, the difference in the overall duration of pubertal growth peak between males and females was only of one and a half month. A literature review reveals insignificant differences in the duration of pubertal growth peak between boys and girls.^31^ Late onset of adolescent growth spurt accompanied by continued post-pubertal increase in mandibular length in boys help explaining large mandibular size and more prevalent Class III jaw relationship in males, as compared to females.^40^ ^,^ ^41^

The results of this study highlight a tendency for Class II subjects towards experiencing pubertal growth peak earlier, and for Class III subjects towards experiencing it later than Class I subjects; however, these differences were of small magnitude and failed to reach the level of statistical significance. Armond et al^42^ evaluated lateral cephalograms of 391 growing children and showed that Class II subjects are twice more likely to enter adolescent growth spurt at an earlier age than Class I subjects. On the other hand, some studies^33^ ^,^ ^37^ report that adolescent growth spurt is likely to start later in Class III subjects, as compared to Class I. Conversely, only a few studies^30^ ^,^ ^31^ ^,^ ^32^ showed statistically insignificant differences in the timings of onset of pubertal growth peak among three classes. The present topic needs further investigation, as the preliminary findings suggest that variations in the timing of pubertal growth peak onset may be related to a variable mandibular morphology in the three skeletal classes.

Our results show that the duration of pubertal growth peak was on average 4.2 months shorter in Class II subjects, as compared to Class I subjects. Salazar-Lazo et al^32^ also showed this difference to be of four months. On the other hand, we found, on average, a 5.9-month longer duration of pubertal growth peak in Class III subjects, as compared to Class I subjects. Studies conducted on South American and Caucasian subjects showed this difference to be of 4.8 and five months, respectively.^30^ ^,^ ^31^ These findings are suggestive that a longer duration of pubertal growth peak may be related to a larger size of the mandible.^30^ ^,^ ^31^ ^,^ ^33^ ^,^ ^37^ On the other hand, a shorter duration of pubertal growth peak may result in early deceleration of mandibular lengthening; thus, resulting in a smaller final size of the mandible.^33^ ^,^ ^37^

The current recommendations endorse CS3 as the ideal time for the initiation of functional jaw orthopedics for the treatment of mandibular deficiency.^4^ Functional appliance therapy is less likely to be successful if commenced in the prepubertal period instead of pubertal growth peak.^5^ In the context of our results and the findings of previous studies,^10^ ^,^ ^33^ ^,^ ^37^ ^,^ ^42^ Class II subjects and girls can be regarded as early maturers, for which commencement of functional jaw orthopedics should be started earlier than usual. Similarly, Class III subjects and boys may be considered late maturers; therefore, they may require treatment with Class III orthopedic appliances to be carried out for a longer period of time until the accelerated phase of adolescent growth spurt is over.

Despite some recent investigations^43^ ^,^ ^44^ showing a weak correlation between CVM and mandibular growth spurt, strong evidence is available in favor of CVM as a good predictor of mandibular growth peak.^4^ ^,^ ^5^ ^,^ ^18^ ^-^ ^20^ ^,^ ^24^ ^-^ ^27^ ^,^ ^45^ ^,^ ^46^ Different studies reported variable levels of validity and reliability of the CVM method ranging from below average to excellent.^47^ ^,^ ^48^ ^,^ ^49^ However, Santiago et al^47^ showed a moderate to high-level of reproducibility of the CVM method in their systematic review using Kappa statistics. A high degree of intra- and interexaminer reliability was found in the current study. Though a recent meta-analysis^25^ shows that the CVM method of Hassel and Farman performs better than Baccetti's method in predicting the overall status of pubertal growth spurt, the later was used because of its proven efficiency in assessing the pubertal growth peak, as shown by longitudinal studies.^28^ ^,^ ^29^ Assessment of craniofacial growth asks for a longitudinal study design as an essential method for reliable results. Longitudinal studies require repeated exposure to X-ray radiations, which has certain ethical limitations. Moreover, a few studies report that variations in skeletal and dental maturation may be related to the vertical facial pattern of the individual.^50^ ^,^ ^51^ In this context, subjects were matched according to the vertical facial pattern by means of SNMP angle and LAFH/TAFH ratio which have been shown to be the most reliable indicators of vertical growth pattern.^52^ Moreover, separate analyses were performed for boys and girls, as required. 

Since the results reported in the present study are derived from cross-sectional data, they may not be the true representative of longitudinal changes. In addition, body height and nutritional status of children is difficult to assess in a retrospective study design. However, the statistical significance of our results is supported by an adequate sample size and highly significant effect sizes for the differences reported in the duration of pubertal growth peak among various groups. Effect sizes, along with probability values, helped us in highlighting the magnitude of differences between males and females and among skeletal classes.^53^ Lastly, the current study used only ANB angle and Downs facial angle to classify subjects. The former lacks the ability to differentiate abnormal growth of the maxilla from that of the mandible, while the later evaluates position of bony chin only with respect to the nasion. Moreover, the reliability of the ANB angle in assessing jaw relationships has been questioned by some authors because of potential erroneous interpretation related to unusual craniofacial morphology and tracing, as well as measurement errors. In this context, a longitudinal study design along with the use of a 3D imaging technique remains as the standard methodology and should be implemented when assessing growth-related changes in the craniofacial skeleton whenever possible.

## CONCLUSIONS

There is no significant difference in the duration of pubertal growth peak between girls and boys. The average duration of pubertal growth peak was found to be 11 months in Class I, seven months in Class II and 17 months in Class III subjects. However, no significant interclass differences were found in the time of pubertal growth peak onset among three skeletal classes. 

A 4-month shorter duration of pubertal growth peak in Class II subjects and a 6-month longer duration of pubertal growth peak in Class III subjects, as compared to Class I subjects, may explain a smaller and a larger increment in mandibular length during pubertal growth peak in Class II and Class III subjects, respectively. However, the validity of these results needs to be endorsed by findings of longitudinal studies. 
